# Neuron-Glia Crosstalk in the Autonomic Nervous System and Its Possible Role in the Progression of Metabolic Syndrome: A New Hypothesis

**DOI:** 10.3389/fphys.2015.00350

**Published:** 2015-12-01

**Authors:** Rodrigo Del Rio, Rodrigo A. Quintanilla, Juan A. Orellana, Mauricio A. Retamal

**Affiliations:** ^1^Centro de Investigación Biomédica, Universidad Autónoma de ChileSantiago, Chile; ^2^Dirección de Investigación, Universidad Científica del SurLima, Perú; ^3^Departamento de Neurología, Escuela de Medicina, Pontificia Universidad Católica de ChileSantiago, Chile; ^4^Centro de Fisiología Celular e Integrativa, Facultad de Medicina. Clínica Alemana Universidad del DesarrolloSantiago, Chile

**Keywords:** glia, connexins, metabolic syndrome, mitochondria, tripartite synapse, hemichannels

## Abstract

Metabolic syndrome (MS) is characterized by the following physiological alterations: increase in abdominal fat, insulin resistance, high concentration of triglycerides, low levels of HDL, high blood pressure, and a generalized inflammatory state. One of the pathophysiological hallmarks of this syndrome is the presence of neurohumoral activation, which involve autonomic imbalance associated to hyperactivation of the sympathetic nervous system. Indeed, enhanced sympathetic drive has been linked to the development of endothelial dysfunction, hypertension, stroke, myocardial infarct, and obstructive sleep apnea. Glial cells, the most abundant cells in the central nervous system, control synaptic transmission, and regulate neuronal function by releasing bioactive molecules called gliotransmitters. Recently, a new family of plasma membrane channels called hemichannels has been described to allow the release of gliotransmitters and modulate neuronal firing rate. Moreover, a growing amount of evidence indicates that uncontrolled hemichannel opening could impair glial cell functions, affecting synaptic transmission and neuronal survival. Given that glial cell functions are disturbed in various metabolic diseases, we hypothesize that progression of MS may relies on hemichannel-dependent impairment of glial-to-neuron communication by a mechanism related to dysfunction of inflammatory response and mitochondrial metabolism of glial cells. In this manuscript, we discuss how glial cells may contribute to the enhanced sympathetic drive observed in MS, and shed light about the possible role of hemichannels in this process.

## Metabolic syndrome and autonomic nervous system imbalance

The metabolic syndrome (MS) is a clinical disorder characterized by the common co-occurrence of several physiological alterations, including increased abdominal fat, elevated fasting glucose, high concentration of triglycerides, low levels of HDL and high blood pressure. People suffering MS are more likely to later developing diabetes mellitus and coronary heart disease, consequently, their life expectancy is reduced (Eckel et al., 2005; Grundy, 2008). This disorder has become a growing health problem that affects millions of people worldwide. Indeed, in United States nearly 50% of people with 60 years or more were estimated to have the metabolic syndrome in 2011–2012 (Aguilar et al., 2015). Up to now, most efforts to understand MS have been focused on the study of peripheral organ malfunction, however, the role of the nervous system on the alterations observed in MS remains unclear.

In the last decade, different groups have proposed that autonomic nervous system (ANS) imbalance may be the hidden factor underlying the progression of different metabolic diseases, including MS (Thayer et al., 2010; Licht et al., 2013; Wulsin et al., 2015). One of the main physiological challenges in the daily life is to keep and maintain the body homeostasis. The ANS conveys sensory afferent information from several territories (e.g., blood vessels, heart, and kidneys) toward nuclei within the central nervous system (CNS), including the medulla and hypothalamus. The sensory inputs are integrated by specific neuronal networks that orchestrate highly coordinated responses to promote adaptive cardiovascular, respiratory, fluid, and energy balance. These functions are complex and requires fine adjustments between the two major branches of the ANS; the sympathetic nervous system (SNS); associated with energy mobilization; and the parasympathetic nervous system (PNS); linked with vegetative and restorative functions. Under physiological conditions, the activities of these branches are in balance. However, when one branch dominates over the other some diseases emerge.

ANS imbalance typically relies on hyperactivation of the SNS and low activity of the PNS, resulting in insulin resistance, altered lipid metabolism, increased blood pressure and endothelial dysfunction (Palatini et al., 2006; Tentolouris et al., 2006; Straznicky et al., 2012; Zucker et al., 2012; Paton et al., 2013; Stern and Filosa, 2013). Indeed, hyperactivation of pre-sympathetic neurons located at the CNS has been pointed out as a key step in the sympatho-excitation observed in MS and further heart failure and diabetes (Li et al., 2008; Zucker et al., 2012; Del Rio et al., 2013; Khoo et al., 2013; Guimaraes et al., 2014; Tremarin et al., 2014; Del Rio, 2015; Moreira et al., 2015; Schlaich et al., 2015). Importantly, evidence from a large cross-sectional study revealed that high sympathetic activity and/or low parasympathetic activity were associated to higher blood pressures, serum triglycerides, serum glucose, and waist circumference (Licht et al., 2010). Neurons that control basal sympathetic activity are located in diverse brain areas, including the paraventricular nucleus of the hypothalamus (PVH), the rostral ventrolateral medulla (RVLM), the spinal cord and the nucleus of the solitary tract (NTS). Among these nuclei, the PVH contains the pre-autonomic neurons that project to the RVLM and spinal cord. At the RVLM, two well-known populations of neurons project toward the spinal cord and other areas, contributing to autonomic regulation (Swanson and Sawchenko, 1983). One population encompassing about of 50–70% of projecting RVLM neurons (C1 group) are glutamatergic but they also synthesize diverse catecholamines, including adrenaline (Guyenet, 2006). Interestingly, non-spinal C1 neurons from the RVLM can innervate the hypothalamus, modulating the excitatory drive to the PVH during baroreceptor activation, a key step in the neural control of circulation (Verberne et al., 1999). Despite the current progress in the field, most of efforts to understand the hyperactivation of SNS during MS have been focused in neurons. Here, we discuss and hypothesize how glial cells and their interaction with neurons at the nuclei that control sympathetic activity could be involved in the pathogenesis and progression of MS.

## General functions of glial cells

In the last two decades, glial cells have emerged as critical players in processing of highly complex information in the CNS. This is particularly true for astrocytes, which create a far-reaching syncytial network that anatomically and functionally communicate neuronal synapses with brain blood vessels (Volterra and Meldolesi, 2005). Through their processes, each astrocyte contact multiple chemical synapses (Oberheim et al., 2006). Thus, astrocytes together with pre- and postsynaptic neuronal structures constitute the “tripartite synapse” (Araque et al., 1999). Embedded in this structure, astrocytes sense neuronal function and respond locally by releasing bioactive molecules termed “gliotransmitters” (e.g., glutamate, ATP, and D-serine) (Perea et al., 2009). In addition, astrocytes also can project specialized terminal processes known as “endfeet” toward capillaries, intracerebral arterioles, and venules; covering about of 99% of abluminal vascular surface (Simard et al., 2003). This complex interaction with neurons and vascular cells facilitate local and long distance astrocytic release of gliotransmitters and vasoactive factors, thereby modulating different neuronal circuits, and networks.

Astrocytes play a crucial role in both gliotransmitter and ionic homeostasis. During high rates of neuronal activity, glutamate and K^+^ accumulated in the cleft are taken up by astrocytes through excitatory amino-acid transporters (EAATs) and inwardly rectifying K^+^ channels or Na^+^/K^+^-pumps, respectively (Allaman et al., 2011). Glutamate and K^+^ once inside of the astrocytes diffuses to neighboring astrocytes and oligodendrocytes via channels known as gap junction channels (GJCs), a process termed “spatial buffering.” Afterwards, glutamate is metabolized to glutamine by glutamine synthetase and released to the extracellular milieu from which it is taken up by neurons and transformed to glutamate or GABA (Allaman et al., 2011). By similar mechanisms astrocytes support metabolic status in neurons. Under physiological conditions, endothelial cells of the blood brain barrier (BBB) take up blood glucose and lactate through GLUT-1 and monocarboxylate transporters (MCTs), respectively. Both lactate and glucose diffuse between adjacent endothelial cells and eventually are taken up by astrocytic and released to the interstitial space (Allaman et al., 2011). Glucose and lactate can diffuse through astrocytes and their gap junctions with neighboring astrocytes to reach relatively distant areas. Glucose can be metabolized to lactate by astrocytes, and both can be released into the extracellular space and taken up by neurons.

Microglia constitute about 5–15% of total cells in the CNS and are essential players of brain innate immune system (Lawson et al., 1990). Originating from peripheral mielomonocytic precursor cells (*fetal macrophages*), microglia populate the brain parenchyma before developmental closure of BBB (Ginhoux et al., 2010). In a healthy brain, microglia exhibit a resting surveillance state (ramified morphology) linked with active exploration of their environment and permanent searching for exogenous or endogenous signals representing a brain threat (Streit, 2001; Kettenmann et al., 2011). When homeostatic balance is altered, resting phenotype of microglia shift to a reactive one, with different degrees of activation depending on nature, intensity and duration of the stimuli (Hanisch, 2002; Block et al., 2007). During brain damage, rather than show a repair-orientated profile, reactive microglia constitute a source of toxic and pro-inflammatory factors (phagocytic morphology), favoring the recruitment of non-resident brain cells involved in the innate and adaptive immune response (Block et al., 2007). In addition to their well-known role on brain immunity and inflammatory response, microglia are now recognized as essential players in the integration and consolidation of neuronal circuits. Various studies have shown that microglia constantly extend toward and retract from synapses, participating in a new range of undiscovered functions, including neuronal surveillance, synapse elimination and regulation of cell death, among others (Tremblay et al., 2010; Schafer et al., 2013; Wake et al., 2013). Indeed, some authors have proposed to shift the current notion of tripartite synapse into a “quad-partite synapse” (Schafer et al., 2013). Interestingly, neurotransmitter release by neurons modifies various aspects of glial cell function, including cellular migration, phagocytosis, intercellular Ca^2+^ wave generation, metabolic coupling, blood flow control and gliotransmitter release among others (Fields and Stevens, 2000; Fields and Stevens-Graham, 2002; Fields and Burnstock, 2006; Inoue et al., 2007). The latter encloses a permanent feedback loop of interactions between neurons and glial cells denominated “neuron-glia crosstalk.”

**Figure 1:**
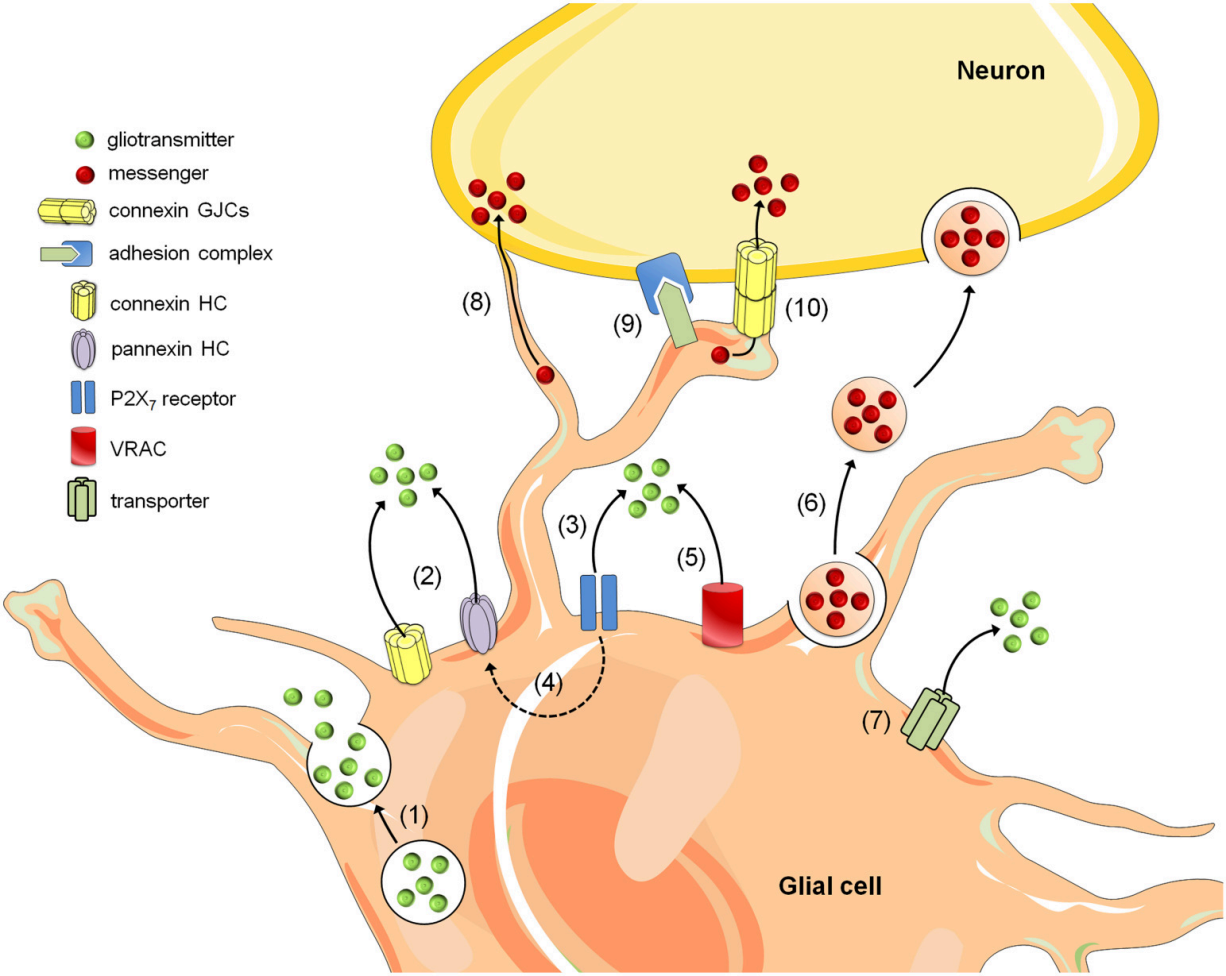
**Mechanisms of glia-to-neuron communication**. Glial cells release gliotransmitters (e.g., glutamate, D-serine, and ATP) through Ca^2+^- and SNARE-dependent exocytosis (1) in addition to the release that occurs through alternative non-exocytotic pathways (see below). Depolarization, reductions in extracellular divalent cation concentrations, increases in intracellular Ca^2+^ and posttranslational modifications might result in the opening of connexin and pannexin hemichannels (HCs) and thus allow the release of gliotransmitters (2). Long-lasting activation of P2X_7_ by ATP might lead to the appearance of large currents and the rapid exchange of large molecules, including the release of gliotransmitters (3). One theory states that P2X_7_ receptor conductance dilates over the time and thereby allows the passage of large molecules; however, another hypothesis states that ATP activate a second non-selective permeabilization pathway (Baroja-Mazo et al., 2013). Recently, it was shown that Panx1 hemichannels might mediate this permeability for large molecules in astrocytes (4) (Iglesias et al., 2009). Additionally, gliotransmitter release may occur through volume-regulated anion channels (VRAC) (5) and different carriers and/or co-transporters acting normally or in reverse (6) (e.g., excitatory amino-acid transporters, the cystine-glutamate antiporter, and the D-serine/chloride co-transporter). Within the last decade, a growing body of evidence has indicated that glial cells can also communicate with neurons via the release of vesicles (e.g., exosomes, microparticles, and apoptotic bodies), containing different cellular messengers (e.g., mRNA, viruses, and organelles) (7). Adjacent glial cells and neurons can communicate directly through F-actin-based transient tubular connections known as tunneling nanotubes (8), via cell-to-cell contacts between membrane-bound ligand molecules and their receptors (9) or aggregates of intercellular channels known as gap junctions, which allow the exchange of small molecules (10).

Gliotransmission is part of the basis of “neuron-glia crosstalk” and multiple mechanisms have been described to mediate gliotransmitter release, including the Ca^2+^-dependent exocytosis (Bezzi et al., 2004; Zhang et al., 2004; Imura et al., 2013), carrier membrane transport (Rossi et al., 2000) and opening of a wide-range of channels encompassing P2X_7_ channels (Duan et al., 2003; Suadicani et al., 2006; Hamilton et al., 2008), volume-regulated anion channels (Kimelberg et al., 1990; Takano et al., 2005; Rudkouskaya et al., 2008; Lee et al., 2010) and connexin hemichannels (Stout et al., 2002; Ye et al., 2003) (Figure 1). Though most studies regarding neuron-glia crosstalk have been focused in gliotransmitter release, in the last decade it has become evident that brain cells can communicate via alternative mechanisms. Among them are those relying on heterotypic glia-to-neuron contacts mediated by homophylic and heterophylic adhesion molecule interactions (Avalos et al., 2009; Sandau et al., 2011) (Figure 1). Importantly, vesicles containing molecules/organelles (e.g., exosomes, microparticles, and apoptotic bodies) have resulted in an new unexpected mechanism of brain cell communication, allowing the exchange of gliotransmitters, organelles, genetic information, proteins, and infectious agents between glial cells and neurons (Frühbeis et al., 2013). Direct astrocyte-to-neuron communication not only occur through GJCs (Fróes et al., 1999; Rozental et al., 2001; Dobrenis et al., 2005), but also via intercellular bridges or long cellular extensions called intercellular nanotubes (Wang et al., 2012) (Figure 1). In the next section, we focused in a specific route of gliotransmitter communication mediated by single membrane channels called “hemichannels.”

## Hemichannels and glia-to-neuron communication

Hemichannels are composed of six protein subunits called connexins (Cxs). The latter encompass a highly conserved protein family encoded by 21 genes in humans and 20 in mice, with orthologs in other vertebrate species (Söhl and Willecke, 2004). For a long time, the pivotal function attributed to hemichannels was to provide the building blocks of GJCs, permitting direct but selective cytoplasmic continuity for ions and molecular exchange between contacting cells (Sáez et al., 2003). Nevertheless, recent studies have shown that hemichannels in “non-junctional” membranes can allow the permeation of ions and small molecules and thus, provide a diffusional route of exchange between the intra- and extracellular milieu (Sáez et al., 2005). Accordingly, hemichannels allow the cellular release of autocrine and paracrine signaling molecules (e.g., ATP, glutathione, glutamate, D-serine, NAD^+^, and PGE_2_) to the extracellular milieu, as well as the entry of other important signaling ions and molecules (e.g., Ca^2+^, cADPR, and glucose) (Retamal et al., 2015). Recently, a new family of three membrane proteins termed pannexins (Panxs 1–3) was shown to form single membrane channels with similar paracrine signaling features of hemichannels (Panchin, 2005). Despite that Cxs and Panxs do not share significant amino acid sequences, both Cx hemichannels and Panx channels (hereinafter referred as Panx for simplicity) exhibit similar membrane topology and oligomerization features.

Astrocytes are characterized by their higher expression of Cx30 and Cx43 (Dermietzel et al., 1989; Batter et al., 1992), but other Cxs such as Cx26, Cx40, Cx45, and Cx46 have been detected in a lesser extent (Scemes et al., 1998; Rouach et al., 2008). Astrocytes also express important levels of Panx1 (Iglesias et al., 2009; Santiago et al., 2011), whereas both Cx43 and Panx1 have demonstrated to form functional channels in astrocytes *in vitro* and *in vivo* (Iglesias et al., 2009; Karpuk et al., 2011; Santiago et al., 2011; Orellana et al., 2015). Furthermore, it has been shown that astrocytic hemichannels are permeable to different molecules (Giaume et al., 2013; Montero and Orellana, 2014), thus allowing the release of ATP (Stout et al., 2002; Anderson et al., 2004; Iglesias et al., 2009; Garré et al., 2010), glutamate (Ye et al., 2003), aspartate (Ye et al., 2003), taurine (Stridh et al., 2008), D-serine (Pan et al., 2015), and glutathione (Rana and Dringen, 2007), as well as the uptake of glucose (Retamal et al., 2007). Up to now, only few studies have documented the expression of functional hemichannels in microglia. Cx32 was the first Cx documented able to form hemichannels in microglia. Pioneering observations by Takeuchi and colleagues, showed that TNF-α induces the release of glutamate (Takeuchi et al., 2006), whereas the expression of functional hemichannels formed by Cx43 and Panx1 also has been reported (Orellana et al., 2011a, 2013a).

Which are the major functions of hemichannels in the brain? In the CNS, hemichannels play different physiological roles including ischemic tolerance or preconditioning (Schock et al., 2008), establishment of adhesive interactions (Cotrina et al., 2008), fear memory consolidation (Stehberg et al., 2012), synaptic transmission (Chever et al., 2014a), glucose sensing (Orellana et al., 2012a; Orellana and Stehberg, 2014), chemoreception (Reyes et al., 2014), BBB permeability (De Bock et al., 2011), and neuronal migration (Elias and Kriegstein, 2008). However, an increasing body of evidence has situated hemichannels as potential regulators of starting and maintaining homeostatic imbalances in diverse brain diseases (Orellana et al., 2009, 2012b, 2013b; Davidson et al., 2013; Takeuchi and Suzumura, 2014; Retamal et al., 2015). Indeed, uncontrolled opening of glial cell hemichannels induced trigger an excessive release of gliotransmitters (e.g., ATP, glutamate, and D-serine) that could be neurotoxic for neurons (Orellana et al., 2012b). Recently was demonstrated that astrocytes or microglia stimulated with amyloid-β (Aβ) peptide exhibit increased Cx43 and Panx1 hemichannel-dependent glutamate and ATP release, which results toxic for hippocampal and cortical neurons (Orellana et al., 2011a). Similarly, follow-up work showed that astrocytes pre-treated with conditioned media from Aβ peptide-treated microglia, release neurotoxic amounts of glutamate and ATP via Cx43 hemichannels when subjected to hypoxia in high glucose containing medium (Orellana et al., 2011b). Importantly, the release of both gliotransmitters reduced neuronal survival via activation of neuronal NMDA/P2X7 receptors and Panx1 (Orellana et al., 2011a,b). How glutamate/ATP affects neuronal hemichannel opening and survival? Most available evidence indicates that neurons express functional hemichannels formed by Panx1 or Cx36 (Thompson et al., 2006; Schock et al., 2008). The opening of Panx1 could occur by protein-protein interactions with activated P2X_7_ receptors (Iglesias et al., 2008) or through increases in [Ca^2+^]_*i*_ or phosphorylation triggered by activation of P2X_7_ or NMDA receptors, respectively (Locovei et al., 2006; Weilinger et al., 2012).

## Relationship between glial cell activation and inflammation/oxidative stress

In numerous brain disorders, glial cells experience a long-lasting process that underlies striking morphological, molecular and functional changes referred as “reactive gliosis” (Block et al., 2007; Pekny and Pekna, 2014). This phenomenon constitutes a graded, multistage glial cell reaction, which counteracts acute damage, restoring homeostasis and limiting brain parenchyma injury. In this stage, astrocytes show hypertrophy and enlargement of the intermediate filament network via upregulation of the glial fibrillary acidic protein (GFAP) and vimentin and re-expression of nestin (Pekny and Pekna, 2014). In addition, a general impairment of glial cell functions has been described such as altered gliotransmission and Ca^2+^ signaling, disturbed mitochondrial dynamics and antioxidant defense, as well as elevated production of nitric oxide (NO) (Block et al., 2007; Pekny and Pekna, 2014). Although reactive gliosis is an adaptive mechanism for protection, when persistent, it can turn into a detrimental response, leading to uncontrolled production of pro-inflammatory cytokines and reactive oxygen species (ROS), which worsens disease progression. At one end, reactive gliosis leads to neuroinflammation mediated by increased levels of IL-1β, IL-6, TNF-α, IL-3, and TGF-β (Block et al., 2007; Pekny and Pekna, 2014). At the other end, elevated levels of inflammatory mediators further cause the secretion of more cytokines and production of more ROS and reactive nitrogen species (RNS) (Mrak and Griffin, 2005; Rosales-Corral et al., 2010). Therefore, redox metabolism regulates cytokine signaling and vice versa, which in some circumstances could create a vicious cycle between oxidative stress and neuroinflammation (Rosales-Corral et al., 2010).

Glial cells use energy and antioxidant power generated by mitochondria to reduce inflammation and support neuronal health (Quintanilla et al., 2012; von Bernhardi and Eugenín, 2012). These glial beneficial properties have been proposed to be mostly based on the metabolism and fusion and fission dynamics of their mitochondria (Hertz et al., 2007; Stephen et al., 2014). Fusion is necessary to ameliorate stress by mixing components of partially damaged mitochondria, whereas fission helps to produce new mitochondria, but it also serves as quality control by allowing the removal of injured mitochondria, by facilitating apoptosis during significant levels of cellular damage (Westermann, 2010). Importantly, diverse studies have revealed a wide-range of defects in mitochondrial dynamics and bioenergetics in glial cells during inflammation (Brown et al., 1995; Almeida et al., 2001; Motori et al., 2013). In fact, reactive astrocytes exhibit increased glycolytic rate and production of ROS (Brown et al., 1995; Almeida et al., 2001), whereas those exposed to pro-inflammatory cytokines, show mitochondrial fragmentation and reduced mitochondrial respiratory capacity (Brown et al., 1995). Apparently, these effects are produced by activation of Drp-1, a GTPase that control mitochondrial elongation in mammalian cells (Hoekstra et al., 2015). In the same context, treatment with IL-1β increases ROS levels in cultured human astrocytes, an effect which is potentiated by IFN-y (Sheng et al., 2013). Similarly, microglia exposed to lipopolysaccharide (LPS), a bacterial pro-inflammatory agent, exhibit an impairment in mitochondrial oxygen consumption (Moss and Bates, 2001; Chénais et al., 2002; Voloboueva et al., 2013) and increased ROS production (Voloboueva et al., 2013). Other studies have explored the use of specific mitochondrial antioxidants to reduce mitochondrial injury induced by pro-inflammatory cytokines in glial cells (Park et al., 2015). Inhibition of mitochondrial-ROS production by treatment with Mito-TEMPO, a specific mitochondrial antioxidant (Park et al., 2015), suppressed the production of mitochondrial and intracellular ROS in LPS-stimulated microglia (Park et al., 2015). Interestingly, treatment with Mito-TEMPO significantly prevented the LPS-induced increase in TNF-α, IL-1β, and IL-6 levels found in microglia (Park et al., 2015). Altogether this evidence indicates that mitochondrial failure could be critical to perpetuate the vicious cycle between oxidative stress and neuroinflammation.

## Reactive gliosis, inflammation, and oxidative stress in the brain during ms

Different studies indicate that most animals subjected to MS models have systemic inflammation and impaired mitochondrial function and redox metabolism (Ando and Fujita, 2009; Litvinova et al., 2015). In the nervous system, animals subjected to different models of MS (e.g., high fat diet, HFD) exhibit an increased number of reactive astrocytes and microglia, as well as elevated levels of pro-inflammatory cytokines, ROS and lipid peroxidation (Thaler et al., 2012; Tomassoni et al., 2013; Gao et al., 2014; Treviño et al., 2015). For instance, 1 day after mice or rats fed a HFD, microglial reactivity increases in the hypothalamus along with the levels of tumor necrosis factor-α (TNF-α) and interleukin-1β (IL-1 β) (Gao et al., 2014). Interestingly, these inflammatory and oxidative responses do not depend on weight gain, since obese leptin receptor mutant db/db and melanocortin receptor 4 knockout mice do not show reactive microglia when fed a standard chow diet (Gao et al., 2014). Moreover, IFN-γ and IL-1β levels are significantly increased in Zucker diabetic fatty rats, predominantly in hippocampal regions near to activated astrocytes and microglia (Hwang et al., 2014). On the other hand, HFD increases brain lipid peroxidation, which is accompanied by increased ROS and mitochondrial depolarization (Ma et al., 2014). In the same animal model, other authors found increased production of NO, lipid peroxidation, impaired glutathione metabolism and mitochondrial failure (Raza et al., 2015). In summary, these data suggest that inflammation and redox imbalance could be key elements in brain dysfunction occurring during MS.

## Could hemichannels contribute to hyperactivation of sympathetic neurons during ms?

Most of the answer to this question relies in how inflammation, oxidative stress and lipids affect the opening of these hemichannels and whether the release of several substances through them could impair normal neuronal excitability (Bennett et al., 2012). Pioneering studies by Takeuchi and colleagues showed that TNF-α treatment could increase the opening of Cx32 hemichannels in microglia, resulting in glutamate release and further neuronal death (Takeuchi et al., 2006). A follow-up study proposed that glutamate released via Cx32 hemichannels play a key role in neuronal damage promoted by experimental autoimmune encephalomyelitis, amyotrophic lateral sclerosis and Alzheimer's disease (Shijie et al., 2009; Takeuchi et al., 2011). On the other hand, Morita and colleagues showed that IL-1β increases astroglial hemichannel opening in culture and brain slices after exposure to a medium lacking divalent cations (Morita et al., 2007). Similarly, IL-1β and TNF-α directly enhances Cx43 hemichannel opening in astrocytes (Retamal et al., 2007); whereas astroglial hemichannel opening evoked by prenatal inflammation is prevented by blocking TNF-α/IL-1β pathways (Avendaño et al., 2015). Different studies have described that IL-1β and TNF-α induce p38 MAPkinase activation in astrocytes (Clerk et al., 1999; Rossa et al., 2006; Mitchell et al., 2007), causing iNOS activation (Gutiérrez-Venegas et al., 2005; Xu et al., 2006) and further NO production (Guan et al., 1997; Badger et al., 1998). Accordingly, upon treatment with NO donors, astrocytes exhibited an increased hemichannel opening associated with S-nitrosylation of Cx43 (Retamal et al., 2006). Similarly, NO is also involved in the increased hemichannel opening and expression of Panx1 observed in neurons subjected to oxygen and glucose deprivation (Zhang et al., 2008). Moreover, opening of hemichannels and microglia subjected to proinflammatory conditions depend on activation of iNOS/NO and p38 MAP kinase pathways (Orellana et al., 2013a; Avendaño et al., 2015). These data suggest that NO could affect the functional state of hemichannels in brain cells exposed to inflammatory conditions, including MS, where iNOS expression is dramatically increased (Ando and Fujita, 2009; Litvinova et al., 2015).

Other focus of attention should be directed to the high levels of fatty acids and different lipids occurring during the progression of MS. HFD induces reactive astrogliosis within days (Calvo-Ochoa et al., 2014; Yeh et al., 2015), which is associated to important changes in hippocampal function and synaptic transmission (Calvo-Ochoa et al., 2014). Recently, Retamal and co-workers showed that unsaturated fatty acids modulate the activity of Cx46 hemichannels in *Xenopus* oocytes (Retamal et al., 2011), whereas linoleic acid also induces Cx43 hemichannel opening in HeLa cells, through a PI3K/Akt/Ca^2+^-dependent pathway (Figueroa et al., 2013). These data suggest that fatty acids modulate hemichannel opening, which could be an additional and interdependent key step along with inflammation and oxidative stress in the activation of sympathetic neurons during the progression of MS.

Another aspect to take into consideration is the high production of free radicals, ROS and RNS in metabolic disorders. A growing body of studies have described that redox potential modulates hemichannel opening in astrocytes (Retamal, 2014). Pioneering studies by Contreras and colleagues showed that Trolox, a free radical scavenger, blocks hemichannel activity induced by metabolic inhibition in cortical astrocytes (Contreras et al., 2002). Later on, a follow-up work demonstrated that dithiothreitol (DTT), a cysteine-reducing agent, reduced astroglial hemichannel activity observed during ischemia-like conditions (Retamal et al., 2006). Interestingly, the response induced by DTT was mimicked by a cell-permeant reduced glutathione ethyl ester (GSH-EE), but not by the non-permeant GSH, suggesting that intracellular cysteines of Cx43 could be oxidized during brain ischemia, affecting hemichannel function (Retamal et al., 2006). In the context of Cx43 hemichannels, it has been proposed that redox potential could modulate them depending on their phosphorylation status, the latter associated to cell damage (Retamal et al., 2007). Accordingly, in astrocytes under physiological conditions (little Cx43 dephosphorylation), DTT increases hemichannel opening, whereas the opposite occurs in astrocytes exposed to long periods of pathological conditions, including inflammation (conspicuous Cx43 dephosphorylation) (Retamal et al., 2006, 2007; Retamal, 2014). All these data suggest that inflammation, lipids and oxidative stress could be the milestones of hemichannel-dependent glial dysfunction during MS. However, the specific cellular mechanism by how glial cell dysfunction could induce sympathetic neurons hyperactivation during metabolic disorders remains to be elucidated. Astrocytes act as modulators of the synapsis by controlling the neuronal postsynaptic excitability in the NST through release of glutamate (Vance et al., 2015). Moreover, it has been proposed that the interaction between astrocytes and neurons in the hypothalamic paraventricular and supraoptic nuclei, both centers involved in the generation of coordinated neurohumoral responses, influence the autonomic response (Stern and Filosa, 2013). Following this line of thought, the optogenetic activation of astrocytes in the RVLM activates pre-sympathetic neurons in an ATP-dependent manner, thus increasing sympathetic renal nerve activity, arterial blood pressure, and heart rate (Marina et al., 2013). Interestingly, physiological function of astrocytic Cx43 and Panx1 channels include regulation of basal and stimulated excitatory synaptic transmission in the hippocampus (Prochnow et al., 2012; Ardiles et al., 2014; Chever et al., 2014a,b), whereas increased opening of astrocytic Cx43 hemichannels evoked by LPS alters excitatory synaptic activity (Abudara et al., 2015). Indeed, recently, Stehberg and colleagues showed that gliotransmmiter release through astroglial Cx43 hemichannels modulates the neuronal activity in the amygdala (Stehberg et al., 2012). This work, showed that microinjection of specific Cx43 hemichannel blocking peptides into the rat's basolateral amygdala abolished the fear memory consolidation (Stehberg et al., 2012). Additionally, recent works demonstrate that hemichannels expressed in astrocytes modulates: (i) human neuronal cortex activity during development (Moore et al., 2014), (ii) basal activity of hippocampal neurons in adult mice (Chever et al., 2014a), and (iii) the inhibitory interneuron activity in response to local hyperexcitability (Torres et al., 2012). Thus, nowadays, the role of astroglial Cx43 hemichannels as neuronal modulators emerges as an ongoing concept in the neuroscience field. At this regard, the uncontrolled release of ATP, glutamate or D-serine via glial cell hemichannels (Ye et al., 2003; Takeuchi et al., 2006; Orellana et al., 2011a,b; Pan et al., 2015) could play a crucial role in hyperactivation of sympathetic system. All these gliotransmitters have showed to modulate synaptic transmission in different brain areas, including the SNS (Guyenet, 2006).

We speculate that a moderate uncontrolled hemichannel opening could raise intracellular free Ca^2+^ concentration in glial cells, leading to altered gliotransmitter release. Supporting this idea, a recent study revealed that glutamate release via astroglial Cx43 hemichannels is associated to impaired excitatory synaptic activity in pyramidal neurons in response to Schaffer's collateral stimulation (Abudara et al., 2015). In this context, it was recently demonstrated that astrocytes respond to physiological changes of the pO2 at the brainstem. Thus, when pO2 is decreased, astrocytes release ATP to the extracellular media, increasing pre-sympathetic neurons activity (Angelova et al., 2015). These findings suggest that astrocytes are metabolic sensors at the brainstem and changes in their metabolism could modulate sympathetic activity through the release of gliotransmmiters. In agreement with this idea, it has been shown that ATP released from astrocytes in the RVLM, increase renal nerve activity, arterial blood pressure, and heart rate (Marina et al., 2013).

Given that an increased hemichannel opening could lead to synaptic malfunctioning and, therefore, to worsening some conditions associated to MS, it could be interesting to analyze other alternatives as well. Thus, in addition to the release of gliotransmitters that potentially affect normal neuronal synapses, astrocytes may also release ascorbate through VSOACs and hemichannels (Wilson et al., 2000; Ahmad and Evans, 2002). Neuronal metabolism under physiological and particularly pathological conditions is highly oxidative (Lai, 1992; Coyle and Puttfarcken, 1993). Astrocytes have large intracellular concentrations of antioxidants, which include reduced glutathione and ascorbate (Wilson, 1997). Neurons can take up ascorbate released from astrocytes, oxidizing it to dehydroascorbate (DHA). Then, DHA is released from neurons through facilitative glucose transporters (GLUTs) (Corti et al., 2010), and further imported by astrocytes via GLUTs, where it is reduced back to ascorbate and once again released to the extracellular media by a pathway that is sensitive to VSOAC inhibitors (Wilson, 1997). Thus, astrocytic ascorbate represents a way of membrane electron transport, in which, reducing equivalents derived from astrocytic metabolism are shared with neurons, as antioxidant support (Lane and Lawen, 2009), as well as means of promoting non-transferrin bound iron uptake by astrocytes, which may also play neuroprotective roles (Lane et al., 2010). Accordingly, Corti and co-workers, proposed that the ascorbate released by astrocytes attenuates glutamate-induced excitotoxicity, oxidative stress and acidosis in neurons (Corti et al., 2010).

At the other end, if the hemichannel activity is very high, an excessive release of ascorbate can be expected. In the brain, copper is used for several important physiological processes (Lutsenko et al., 2010; Scheiber et al., 2014). However, changes in copper homeostasis have been correlated to development of some neurodegenerative diseases (Scheiber et al., 2014). This is so because ascorbate can increase copper accumulation in astrocytes (Scheiber et al., 2010a,b), which -at high concentrations- is toxic for them (Bulcke et al., 2015), as well as for neurons (Scheiber et al., 2014). A massive ascorbate release from astrocytes can be associated to an increase of copper associated to neuronal death. Interestingly, in Bulcker's work, they reported that cell loss induced by copper/ascorbate was correlated with increased permeability to propidium iodide, which is a fluorescent dye extensively used to measure hemichannel opening in several cell types (Ebihara et al., 2011; Shahidullah and Delamere, 2014; Mandal et al., 2015). We suggest that moderate increase of hemichannel opening observed during MS could lead to synaptic malfunctioning due to a massive release of glio transmitters, but also can help neuronal survival. In addition, massive hemichannel activity could lead to both neuronal and glial cell death, due to excessive glutamate release and overload of copper in astrocytes. Since this idea has not been confirmed in MS yet, future studies are needed focusing on the role of astrocytes as neuron protectors during MS.

## Future directions

**Figure 2:**
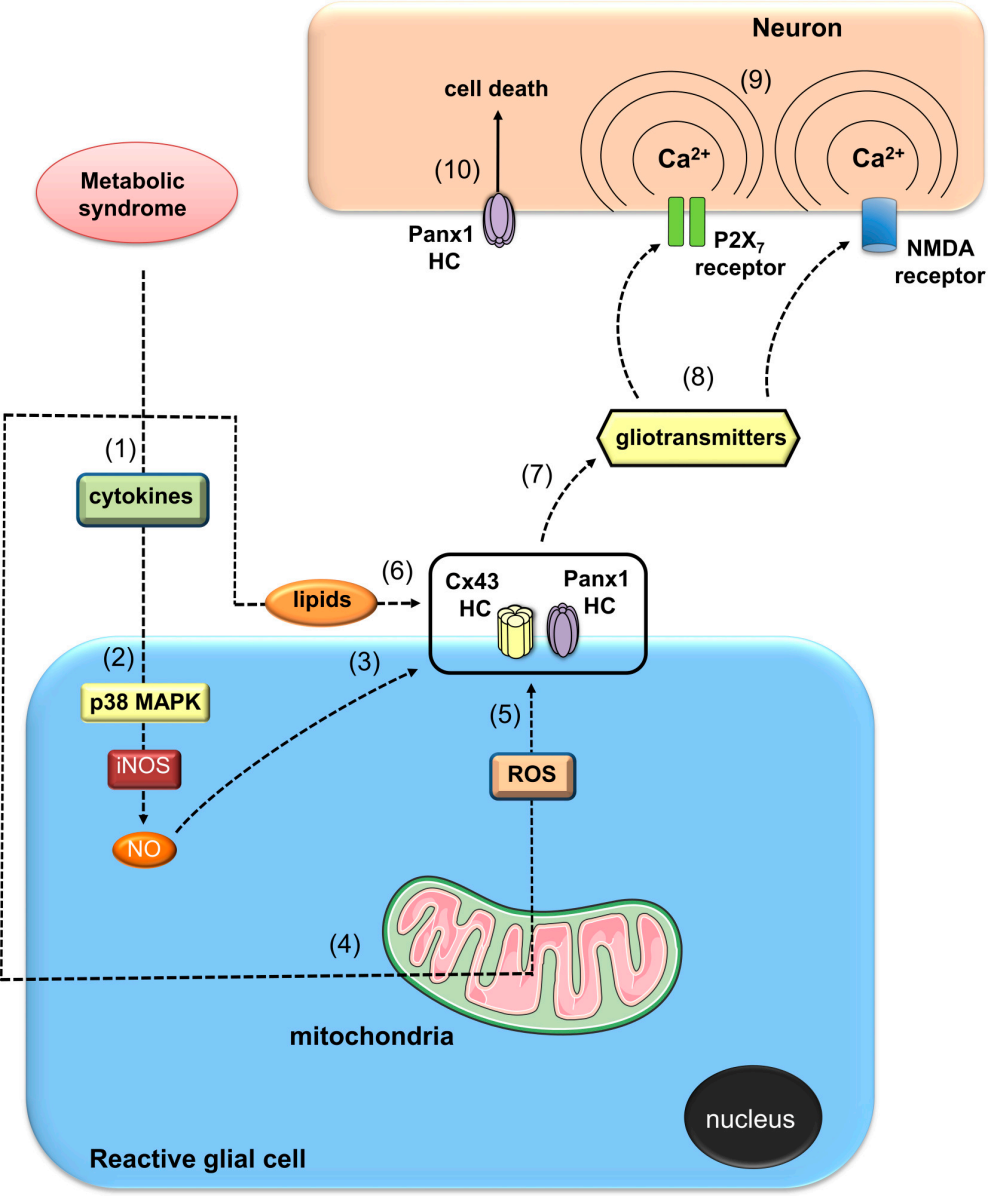
**Possible actions of metabolic syndrome on glia-to-neuron communication mediated by hemichannels**. Metabolic syndrome (MS) may induce a generalized inflammatory state that could affect the nervous system (1). In this context, autocrine/paracrine release of pro-inflammatory cytokines (e.g., IL-1β and TNF-α) by reactive glial cells could lead to the activation of a p38MAPK/iNOS-dependent pathway and further production of nitric oxide (NO) (2). NO could cause the nitrosilation of Cx43, resulting in opening of Cx43 glial cell hemichannels (3). Alternatively, for an unknown mechanism, NO could increase the activity of Panx1 hemichannels. Along with the systemic inflammatory state, MS impairs mitochondrial function in glial cells, leading to redox potential imbalance and subsequent uncontrolled production of reactive oxygen species (ROS) (4). Modulation of oxidative status of Cx43 and/or Panx1 hemichannels by ROS could increase their activity (5). High levels of triglycerides and fatty acids during the progression of MS could directly enhance the opening of hemichannels in glial cells (6). In addition, paracrine release of gliotransmitters through glial cell hemichannels (e.g., ATP, glutamate, D-serine) (7) could act on neighboring or distant neurons, resulting in the activation of P2X_7_ and NMDA receptors (8). The latter increase levels of [Ca^2+^]_*i*_, (9) and thereof the activity of neuronal Panx1 channels, resulting in neuronal function impairment and cell death (10).

Until this point we discussed the possible mechanism that associates glial cell hemichannel opening with the increased sympathetic activation observed during the MS. This hypothesis could plausible if hemichannel opening increases until certain (unknown) level. However, what about if hemichannel activity increase even more? The most obvious suggestion is that neuronal function and synaptic transmission will be compromised, resulting in further production of neuropathies (Retamal et al., 2015). It is well known that metabolic-associated diseases can produce the appearance of neuropathies (Kim and Feldman, 2012; D'Amico and Bertini, 2013). One possibility is that gliotransmitters released from glial cells due to hemichannel opening become neurotoxic, as has been recently demonstrated (Orellana et al., 2011a,b; Avendaño et al., 2015). In summary, we propose that under MS a positive feedback loop can be generated between reactive gliosis, inflammation, mitochondrial dysfunction and hemichannel opening (Figure 2). The latter may contribute to the autonomic imbalance at early stages of MS specifically through a glial cell dependent modulation of sympathetic neuron activity in the brainstem. Importantly, as the disease progress, development of neuropathies could take place mainly associated with the neurotoxic consequence of a massive opening of hemichannels.

### Conflict of interest statement

The authors declare that the research was conducted in the absence of any commercial or financial relationships that could be construed as a potential conflict of interest.
